# Genetic diversity of the Griffon vulture population in Serbia and its importance for conservation efforts in the Balkans

**DOI:** 10.1038/s41598-020-77342-1

**Published:** 2020-11-23

**Authors:** Slobodan Davidović, Mihailo Jelić, Saša Marinković, Milica Mihajlović, Vanja Tanasić, Irena Hribšek, Goran Sušić, Milan Dragićević, Marina Stamenković-Radak

**Affiliations:** 1 Department of Genetics of Populations and Ecogenotoxicology, Institute for Biological Research “Siniša Stanković” – National Institute of the Republic of Serbia, University of Belgrade, Bulevar Despota Stefana 142, 11060 Belgrade, Serbia; 2 Faculty of Biology, University of Belgrade, Studentski trg 16, 11000 Belgrade, Serbia; 3 Department of Ecology, Institute for Biological Research “Siniša Stanković” – National Institute of Republic of Serbia, University of Belgrade, Bulevar Despota Stefana 142, 11060 Belgrade, Serbia; 4 Center for Forensic and Applied Molecular Genetics, Faculty of Biology, University of Belgrade, Studentski trg 16, 11000 Belgrade, Serbia; 5 Birds of Prey Protection Foundation, Bulevar Despota Stefana 142, 11060 Belgrade, Serbia; 6 Ornithological Station Rijeka, Croatian Academy of Sciences and Arts, Ružićeva 5/2, 51000 Rijeka, Croatia; 7 Department of Plant Physiology, Institute for Biological Research “Siniša Stanković” – National Institute of the Republic of Serbia, University of Belgrade, Bulevar Despota Stefana 142, 11060 Belgrade, Serbia

**Keywords:** Genetic variation, Genetic variation

## Abstract

The Griffon vulture was once a widespread species across the region of Southeast Europe, but it is now endangered and in some parts is completely extinct. In the Balkan Peninsula the largest Griffon vulture inland population inhabits the territory of Serbia. We present, for the first time, the genetic data of this valuable population that could be a source for future reintroduction programs planned in South-eastern Europe. To characterize the genetic structure of this population we used microsatellite markers from ten loci. Blood samples were collected from 57 chicks directly in the nests during the ongoing monitoring program. We performed a comparative analysis of the obtained data with the existing data from three native populations from French Pyrenees, Croatia, and Israel. We have assessed the genetic differentiation between different native populations and determined the existence of two genetic clusters that differentiate the populations from the Balkan and Iberian Peninsulas. Furthermore, we analysed whether the recent bottleneck events influenced the genetic structure of the populations studied, and we found that all native populations experienced a recent bottleneck event, and that the population of Israel was the least affected. Nevertheless, the parameters of genetic diversity suggest that all analysed populations have retained a similar level of genetic diversity and that the Griffon vulture population from Serbia exhibits the highest value for private alleles. The results of this study suggest that the Griffon vulture populations of the Balkan Peninsula are genetically differentiated from the populations of the Iberian Peninsula, which is an important information for future reintroduction strategies.

## Introduction

Eurasian Griffon vulture (*Gyps fulvus fulvus* Hablizl, 1783) (hereafter Griffon vulture) belongs to the scavenger guild composed of 8 species distributed in the Old World^1^. The IUCN Red List status of African-Eurasian vultures has witnessed a drastic population decline in recent years and the majority of species were listed as Critically Endangered^2^. Griffon vulture species is an object of several international conservation conventions and directives. It is currently listed as a priority species for protection by the European Union (Annex I of the European Birds Directive, as Eurasian Griffon Vultures *Gyps fulvus*), Multi-species Action Plan for avian scavenger MsAP^2^, Convention on Biological Diversity, Bonn Convention, Bern Convention, CITES and BirdLife international. Thanks to the conservation efforts, Griffon vulture is today considered among the least concerns globally^3^. Europe alone is the home to more than 32,400–34,400 (Birdlife International 2017) pairs of birds, but although this is a large number of individuals, only 10% of all European Griffon vultures live outside the Iberian Peninsula^4,5^.

Although Griffon vulture populations have been recovered in Southwest Europe, the populations are still declining in many parts of Southeast Europe (Balkan Peninsula), Caucasus region, the Middle East and North Africa^6–8^. The vulture populations of the Balkan Peninsula reached a critical conservation status at the end of the twentieth and the beginning of the twenty-first century mainly due to poisoning. As a consequence, in many Balkan countries the populations drastically declined to become highly threatened in some (continental Greece and Macedonia) and completely extinct in others (Albania, Bosnia & Herzegovina, Montenegro and Romania). Today the only viable populations inhabit the territories of Serbia, Croatia, Crete and Bulgaria^9^. Although a Mediterranean species, it can be found in mainland enclaves that are located in gorges and canyons of the central Balkan Peninsula, which represent biodiversity hotspots^10,11^. Unfortunately, the range of this species has almost completely diminished and in Serbia they can be found only in two regions of its western part, in the gorges of the Drina river tributaries^12^.

The Griffon vulture population in Serbia, similarly to many Balkan countries, experienced a rapid demographic decline starting from the mid-twentieth century, mainly due to the mass poisoning of the birds and the implementation of new veterinary measures that prohibited the deposition of dead animals in nature^13^. This negative demographic trend lasted until the ‘90s when Griffon vulture population decreased to only 10 breeding pairs^12^. Thanks to the successful conservation efforts, Griffon vulture population in Serbia is now the largest in the Balkan Peninsula, and constantly increasing. During the 2019^14^ census 260 pairs of birds were detected, out of which 164 were breeding pairs. Although the conservation efforts of Griffon vulture in Serbia seem to represent a success story, no genetic analysis has been performed so far, and there are no data of its overall genetic variability.

Genetic diversity analysis is of great importance in modern-day conservation, and without knowing the genetic status of the population it is hard to implement proper conservation measures and secure long term survival of the total population in the nowadays fast-changing environment and habitat fragmentation^15–19^. Because of this, IUCN has recognized genetic diversity as one of the three forms of biodiversity that deserve conservation, along with species and ecosystem diversity^20^. In territories where a species is extinct, like it is the case with the Griffon vulture in parts of the Balkan Peninsula, the programs of reintroduction sometimes are the only way of restoring the population^21^. These programs have to pay close attention to the genetic structure of the source population and founding individuals, in order to avoid post-reintroduction genetic drift that could have negative consequences^22–24^. The problem of genetic diversity and reintroduction of the Griffon vulture was addressed in the paper of Le Gouar^25^. They demonstrated that a proper choice of individuals used for the founding population can help to retain overall genetic diversity.

Due to the high mobility of this species and known migrations undertaken by immature birds, it was believed that Griffon vulture populations represented a uniform population within its European range, with low genetic differentiation, as suggested by genetic analyses^25,26^. The proposed genetic uniformity of Griffon vulture populations is contrary to the high levels of among-population differentiation that was observed for other vulture species like the Bearded vulture, *Gypaetus barbatus*^27^. It was also demonstrated that the geographical barriers can cause differentiation in the populations of Cooper’s Hawk, *Accipiter cooperii*^28^, as well as between the Bearded vulture populations of the Pyrenees and its reintroduced populations in the Alps^29^. Interestingly, isolation on islands was also identified as the cause of genetic differentiation for the populations of Egyptian vulture, *Neophron percnopterus*^30^ and Griffon vulture populations of Sardinia, Crete and Cyprus^18^. The strong phylogeographic pattern was detected within the populations of Cinereous vulture, *Aegypius monachus*^31,32^, which was explained by the philopatric behaviour that can cause a limited gene flow among populations^27,32^. Similarly, subtle population structuring detected in the Cape vulture, *Gyps coprotheres*, populations in South Africa could also be assigned to regional natal philopatry^33^. Given the fact that Griffon vulture, Cinereous vulture and Cape vulture share many behavioural similarities including the philopatric behaviour it could be possible that some level of genetic differentiation could be observed between the geographically distant Griffon vulture populations as well.

In this paper we present for the first time genetic diversity data obtained from the largest and most viable Griffon vulture population that inhabits the part of the Balkan Peninsula with a continental climate. Depending on the genetic structure, it could potentially be the appropriate source population for future reintroduction programs^34^ that are planned in Eastern Serbia and other parts of the Balkan Peninsula. We present a comparative analysis of the obtained data and those published previously on native populations of the Griffon vulture from Croatia, Israel and the Pyrenees in France^25^. Inclusion of the biggest inland Griffon vulture population from the Balkan Peninsula should improve the results of the comparative analysis which will allow us to determine if there is a detectable level of genetic differentiation between geographically distant populations, a pattern that was observed for the Cape vulture and the Cinereous vulture a species with similar behavioural traits as the Griffon vulture.

## Results

### Parameters of genetic diversity

Parameters of genetic diversity per population are presented in Table 1, whereas the parameters of genetic diversity per locus per population are presented in Tables S1, S2. The obtained results demonstrated lack of polymorphism for locus BV13 in the native Griffon vulture populations inhabiting Serbia and Croatia, as well as in the introduced individuals in Navacelles population (Table S1). All other loci were polymorphic in all analysed populations, and the most polymorphic locus overall was BV12, with the highest number of alleles and greatest allelic range (Table S1). The average number of alleles per locus varied from 2.0 to 13.4 (Table S2) while the average number of alleles per population varied from 4.8 to 6.7 (Table 1 and Table S2). Among native populations the highest number of alleles as well as the highest mean number of alleles based on minimal sample size was detected in the Griffon vulture population inhabiting Israel (Table 1 and Table S1). Considering the mean number of private alleles based on minimal sample size the highest number among native populations was observed in Serbia, although the actual number of detected private alleles was highest in Israel and Causses (Table 1). The average effective number of alleles was similar in all native populations and it varied from the lowest detected in the Pyrenees (3.36) to the highest detected in Israel (3.93) (Table 1). The effective number of alleles per locus per population is presented in Table S1. Values for the observed (H_O_) and expected (H_E_) heterozygosity were found to be very close in all populations, ranging from 0.53 to 0.60 for H_O_ and from 0.55 to 0.60 for H_E_. No deviation from Hardy–Weinberg equilibrium was detected in any of the analysed populations (Table S1). The Wilcoxon signed-rank test before the Bonferroni correction demonstrated that all native populations, except the one in Israel, exhibit significant heterozygosity excesses. After the correction the population from Serbia was also without significant heterozygosity excesses (Table 1). The values for G–W index were high for all analysed populations and, among the native populations, the one from Israel exhibited the highest value. The mean number of pairwise differences among native populations was the highest in the Pyrenees (Table 1). The average number of pairwise differences between and within populations is visualized in Fig. S1 together with Nei’s distances. The lowest value for the RMP was detected in the population of the Pyrenees (Table 1). The population with the lowest number of loci with linkage disequilibrium was the population from Israel and out of all native populations the one in the Pyrenees had the highest number of loci with linkage disequilibrium (Table S3). Estimated effective population sizes for all analysed populations are presented in Table 1 and Table S4.

**Table 1:** Genetic diversity parameters, effective population size, Wilcoxon signed-rank test, and Garza–Williamson index determined per native and introduced populations of *G. fulvus*.

Population	N	A	Am	Ap	Ar	Ae	H_O_	H_E_	MPD	RMP	Ne	Wilcox p	G–W
**Native**													
Serbia	57	5.6	3.69	2	0.26	3.43	0.57	0.57	5.66	0.009	294.9	0.05468	0.82
Croatia	37	5.4	3.78	1	0.12	3.73	0.56	0.59	2.91	0.014	92.6	**0.04104**	0.89
Israel	33	6.4	4.05	3	0.19	3.93	0.53	0.56	3.61	0.015	∞	0.27540	0.92
Pyrenees	81	5.9	3.77	1	0.08	3.36	0.59	0.59	5.79	0.006	228.1	**0.00441**	0.89
**Introduced**													
Baronnies	90	6.3	3.71	1	0.06	3.36	0.59	0.58	4.13	0.006	195.4	0.06105	0.89
Causses	86	6.7	3.98	3	0.16	3.68	0.60	0.60	6.02	0.006	102.9	**0.03904**	0.85
Verdon	41	5.5	3.51	0	0.04	2.97	0.53	0.55	4.70	0.012	207.8	0.18750	0.91
Navacelles	15	4.8	3.74	0	0.11	3.15	0.53	0.55	1.71	0.033	79.1	0.37500	0.93
Diois	45	5.4	3.64	0	0.06	3.14	0.54	0.56	4.54	0.012	102.9	**0.03416**	0.92

Mean values are presented.

N, number of genotyped individuals, A, mean number of alleles, Am, number of alleles based on a minimal sample size of 12 diploid individuals, Ap, number of private alleles detected in the population, Ar, number of private alleles based on a sample of 12 diploid individuals, Ae, mean effective number of alleles, H_O_, observed heterozygosity, H_E_, expected heterozygosity, MPD, mean number of pairwise differences, RMP, random match probability, Ne, effective population size, *Wilcox. p*, *p* value corrected by sequential Bonferroni test obtained by the Wilcoxon signed-rank test (*p* < 0.05, the significant *p* value is bolded), G–W Garza–Williamson index.

### Differentiation of analysed Griffon vulture populations

The AMOVA performed across all loci revealed that 2.24% of the genetic variance can be contributed to the variation among the populations while the rest of genetic variance can be explained with the variation within populations (Table 2). The AMOVA performed on different groups of populations showed the existence of generally low values of genetic variance among the groups of populations and the highest observed value, 3.85%, was observed between the group of populations from the Balkan Peninsula (Serbia and Croatia) and the group of populations derived from the Iberian Peninsula (the Pyrenees with reintroduced populations from Baronnies, Causses, Verdon, Navacelles and Diois) (Table S5). The lowest observed value of genetic variance between the groups of analysed populations was observed between the group of populations derived from the Iberian Peninsula (Table S5).

**Table 2:** Outcomes of AMOVA analysis based on the variability of 10 autosomal loci for 9 analyzed populations.

Source of variation	*df*	Sum of squares	Variance components	Percentage of variation
Among populations	8	62.223	0.05236	2.24 (*p* = 0.00)
Within populations	961	2195.802	2.28491	97.76
Total	969	2258.025	2.33727	

**Figure 1:**
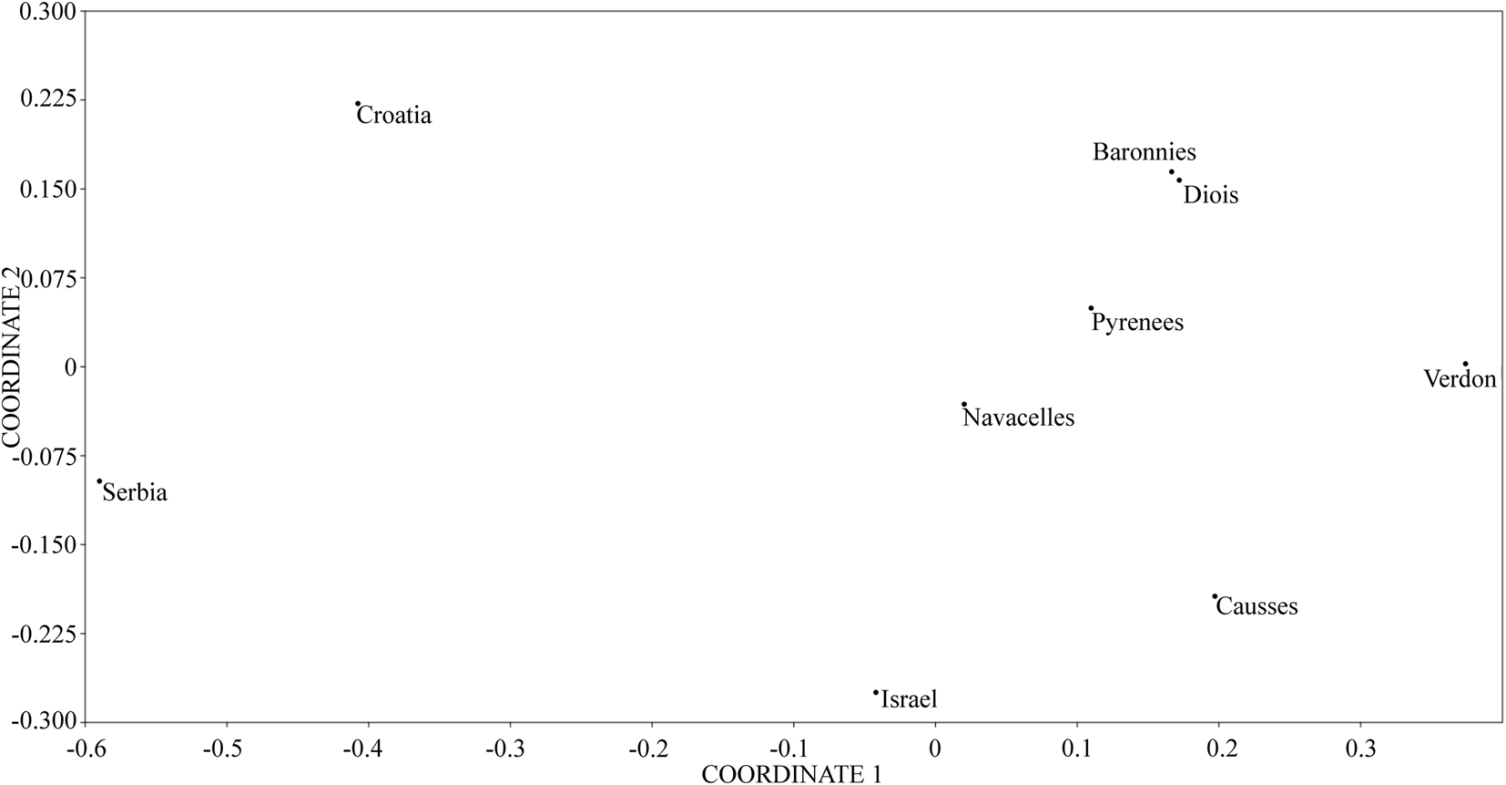
Non-metric multidimensional scaling plot of *F*_*ST*_ distances between the *G. fulvus* population of Serbia and other native and introduced *G. fulvus* populations based on the analysis of 10 microsatellite loci. The goodness of fit is expressed with the stress value which is 0.1644 for this data set. Population pairwise *F*_*ST*_ values are presented in Table 3.

The pairwise population *F*_*ST*_ values between the Griffon vulture population of Serbia and other analysed populations are generally low but statistically significant for all pairs of native populations (Table 3). Pairwise population *F*_*ST*_ is visualized in Fig. S2 and visualization by MDS plot shows the positioning of populations in two dimensions (Fig. 1). Griffon vulture populations of Serbia and Croatia occupy outlying position compared to other populations and together they form a distinct cluster. The Griffon vulture population from Israel is positioned close to the population of the Pyrenees, but between Pyrenees and populations from the Balkan Peninsula. In addition, all introduced populations cluster together with the native population of the Pyrenees from which they originate.

**Table 3:** Pairwise population *F*_*ST*_ (below diagonal) and *F*_*ST*_* p* values (above diagonal) between the populations based on the variability of 10 microsatellite loci in 9 different *G. fulvus* populations.

	Serbia	Croatia	Israel	Pyrenees	Baronnies	Causses	Verdon	Navacelles	Diois
Serbia		**0.00420**	**0.00000**	**0.00000**	**0.00000**	**0.00000**	**0.00000**	0.09659	**0.00000**
Croatia	**0.02121**		**0.00000**	**0.00000**	**0.00000**	**0.00000**	**0.00000**	0.35740	**0.00000**
Israel	**0.04670**	**0.02908**		**0.00800**	**0.00000**	0.07210	**0.00230**	1.00000	**0.03168**
Pyrenees	**0.04565**	**0.02699**	**0.01619**		1.00000	**0.00220**	0.22692	0.99990	1.00000
Baronnies	**0.05299**	**0.03334**	**0.01851**	0.00140		**0.00950**	0.05790	1.00000	1.00000
Causses	**0.06293**	**0.04542**	0.01036	**0.01121**	**0.00848**		**0.01062**	1.00000	**0.02193**
Verdon	**0.05622**	**0.05708**	**0.02232**	0.00727	0.01012	**0.01396**		1.00000	0.34309
Navacelles	0.02077	0.01599	0.00358	− 0.01699	− 0.01339	− 0.01270	0.00785		1.00000
Diois	**0.05616**	**0.04808**	**0.01609**	0.00051	− 0.00023	**0.01119**	0.00810	− 0.01202	

Significant *F*_*ST*_ values (*p* ≤ 0.05; corrected using sequential Bonferroni test) are in bold letters.

**Figure 2:**
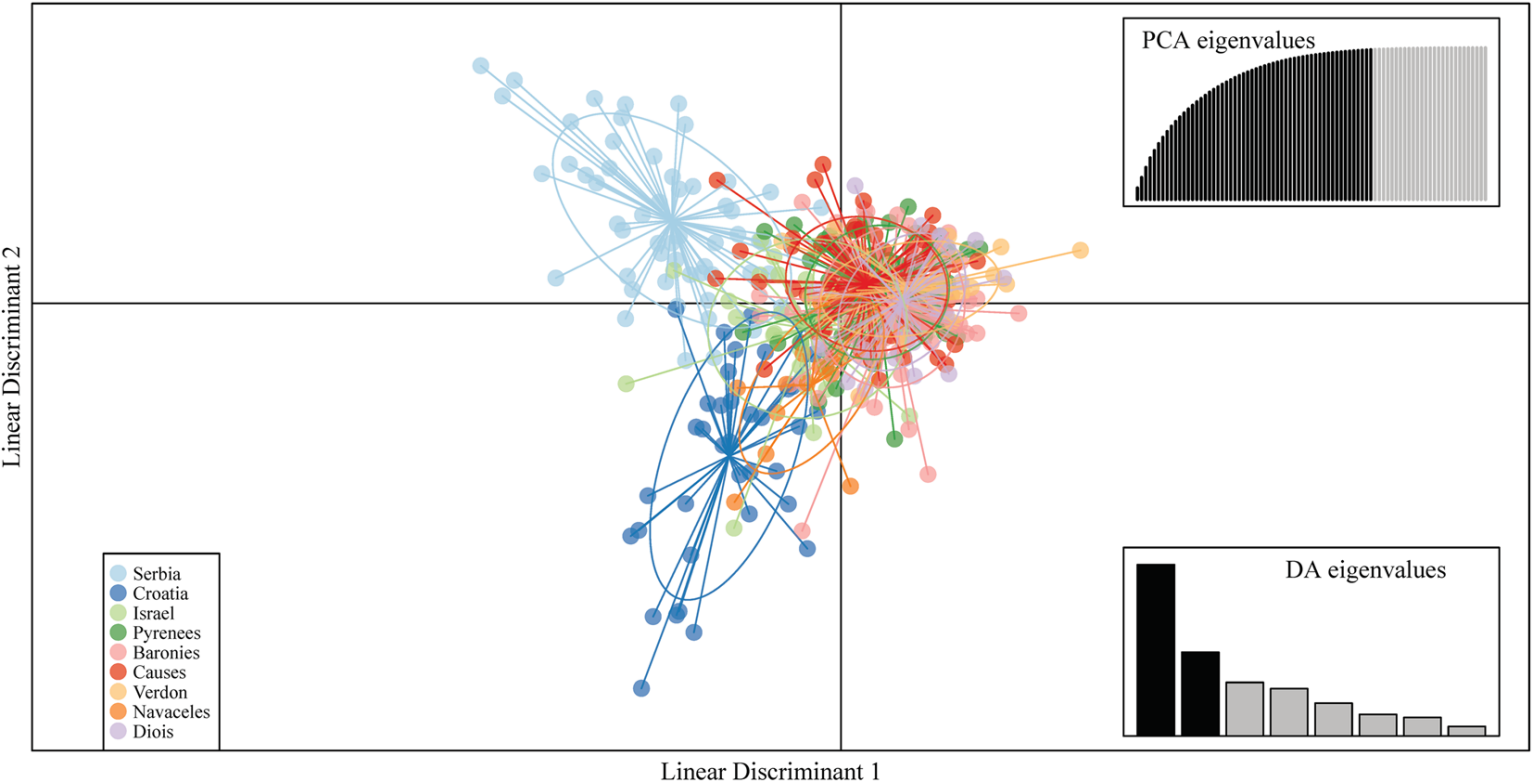
Discriminant analysis of principal components in which LDA was performed on the first 56 PCs (out of 83 PCs since there were a total of 83 observed alleles) which cumulatively conserve 98.7% of the total variance. The first and second lienar discriminant are presented in the plot.

Analysis performed by the DAPC method (Fig. 2) showed that populations cluster in a pattern similar to the one presented in Fig. 1. The birds from Serbia and Croatia form two different clusters of their own, while the cluster that consists of the birds from Israel occupies an intermediary position between all the clusters. The birds from the Pyrenees and the birds from introduced populations are grouped in cluster overlaps, indicating the similarity of these populations. The populations of Baronies, Verdon and Diois contain the birds of Spanish origin, and these birds were used as the sample population representative for Spain in Le Gouar^25^. Additionally, in the same paper, the authors decided to pool the data for French Pyrenees (Ossau) and Spain, since there were no detectable genetic differences between these two populations. These results indicate that the population of the Pyrenees, together with the reintroduced populations, can be used to represent the population of the Iberian Peninsula.

**Figure 3:**
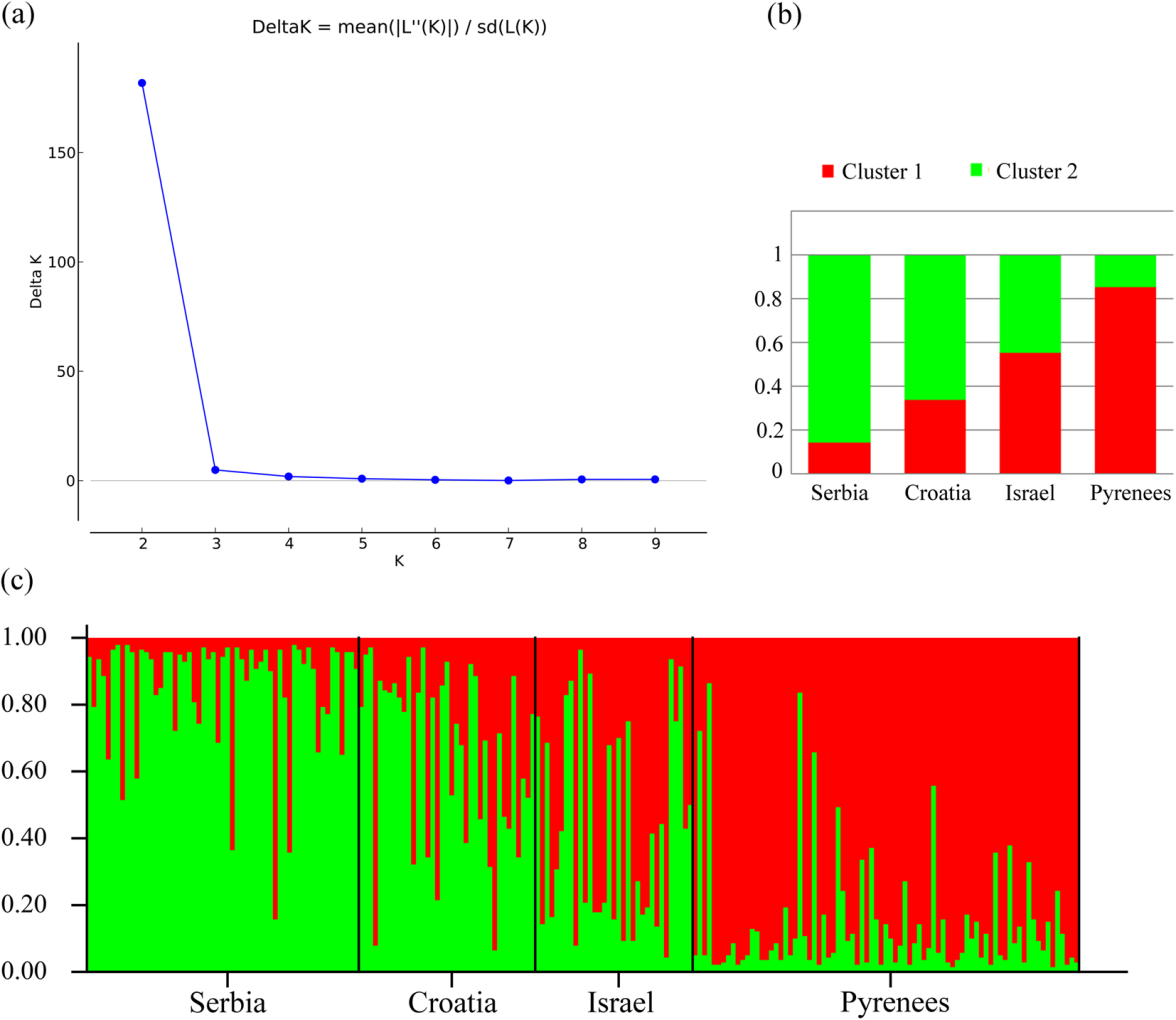
(**a**) Delta *K* values for the assumed number of genetic clusters. (**b**) Proportions of inferred STRUCTURE clusters (*K* = 2) from the native populations. (**c**) Proportions of the inferred STRUCTURE clusters (*K* = 2) from the individuals.

STRUCTURE Harvester showed that *K* = 2 is the most likely scenario (Fig. 3a and Fig. S3). The distribution of two genetic clusters shows that in the Balkans (Serbia and Croatia) the second cluster is dominant, which is opposite from the Pyrenees where the first cluster is dominant, while the population that inhabits the Middle East (Israel) has almost equal contributions from both genetic clusters (Fig. 3b,c). STRUCTURE analysis was performed on native and introduced populations as well, and the results are shown in Fig. S4.

## Discussion

In the present paper we present for the first time genetic data of the Griffon vulture population from Serbia that inhabits parts of the Balkan Peninsula with a continental climate. We have also performed a comparative analysis with the data obtained on the Griffon vulture populations from Croatia, Israel (Mediterranean climate), and the Pyrenees in France, published in Le Gouar^25^.

The endangered Griffon vulture species of the Balkan Peninsula is the last inland population adapted to the continental climate. It could thus be an important source for the reintroduction programs in Southeast Europe. In the present paper we have assessed the genetic variability using the highly variable microsatellite loci described and developed for conservation and reintroduction programs^35,36^. Presented data demonstrate that Griffon vulture population from Serbia exhibits similar values of genetic diversity as other native populations. Similar values of the parameters of genetic diversity were observed for the Griffon vulture populations of Cyprus and Spain^26^ although a slightly different set of microsatellite loci were used in that study. Compared to other endangered vulture species, the Griffon vulture population of Serbia exhibits higher values of genetic diversity than the ones observed in the populations of the Indian vulture (*G. indicus*) from Pakistan^26^, Cape vulture (*G. coprotheres*) from South Africa^33^, Cinereous vulture (*A. monachus*) from Spain, Georgia, Armenia and Kazakhstan^32^, and White-tailed eagles (*Haliaeetus albicilla*) from Norway, Estonia, and Germany^37^. Interestingly the population of Griffon vulture from Serbia exhibits similar values for parameters of genetic diversity when compared with the populations of the Bearded vulture (*Gypateus barbatus*) from the Pyrenees^38^, White-rumped vulture (*G. bengalensis*) from Pakistan^26^, and the Golden eagle (*Aquila chrysaetos canadensis*) from North America^39^, while it exhibits lower values in comparison to the White-backed vulture (*G. africanus*) from Namibia^26^. Although the population of Griffon vulture from Serbia experienced a serious bottleneck during the last decade of the twentieth century, it retained its rich genetic diversity which can be observed even when compared to the biggest and historically most stable population of Europe, the population from the Iberian Peninsula i.e. Pyrenees^40^. This finding corresponds with the observation that the similar long-lived species like White-tailed eagles retained relatively high levels of genetic diversity even after they had experienced a bottleneck event^37^. Some parameters, like the number of private alleles and the mean effective number of alleles, even suggest that the Balkan Peninsula, i.e. populations from Croatia and Serbia, exhibit higher genetic diversity than the ones observed in the Pyrenees. The highest number of private alleles based on a sample of 12 individuals was detected in the Griffon vulture population of Serbia, suggesting that this population could act as an important source of genetic variation^34^.

Considering the bottleneck in the Griffon vulture population of Serbia, the observed G–W index is the lowest observed among all the populations analysed, but is still relatively high and close to the observed values for other analysed native and introduced populations. According to the G–W index, the populations with the values closest to value 1 are considered to be the most stable populations, which is, according to our analysis, the population of Israel. Wilcoxon test, which evaluates the probability of recent bottleneck events based on heterozygosity excess, even suggests that the highest probability for this event is detected for the population of the Pyrenees, and not the population of Serbia. In addition, even the linkage disequilibrium between the pairs of loci suggests a higher probability of the recent bottleneck event for the population of the Pyrenees than the population of Serbia. It has been shown that the reduction in population size leads to a non-random association of alleles for unlinked loci^41–43^ and, further, that the detection of linkage disequilibrium for unlinked loci suggests a recent reduction in effective population size^44^. The effective population sizes estimated for the analysed populations showed that Israel has the biggest population, followed by Serbia and the Pyrenees.

In addition, the discrepancy between the expected and observed bottleneck effects for the population of Serbia can be explained by possible immigration of the birds from nearby related colonies/populations. It is possible that after war conflicts in the region in the early ’90 s the four known Griffon vulture colonies in Eastern Herzegovina (Bosnia and Herzegovina) didn’t truly disappear, instead, they might have resettled in Serbia^45,46^ forming a new colony in 1995 in the Mileševka Gorge^45,46^. These birds might have helped to maintain the high genetic diversity in the population of Serbia, thus reversing the negative effects of the previous rapid population decline.

Population structure analysis revealed the existence of two genetic clusters, and the distribution of these clusters can clearly distinguish two groups that exhibit different proportions, with the addition of one admixed population. Populations from Serbia and Croatia form one group specific to the Balkan Peninsula, while another group would be specific to the Pyrenees, and all the populations derived from the Iberian Peninsula (Fig. 3 and Fig. S4). The third group, i.e. the admixed population, with almost equal proportions of both genetic clusters, is the Israeli population from the Middle East. In addition, all introduced populations are remarkably similar, almost identical, to the population from the Pyrenees. This is expected, since all introduced populations are formed from the birds that are caught and brought from the Spanish and French sides of the Pyrenees^25^. This confirms that, although all introduced populations exhibit high genetic variations, they form one continuous and uniform population with their source population (Fig. S4), which is a pattern also observed in Le Gouar^25^.

Additionally, the *F*_*ST*_ values, as well as MDS, clearly demonstrate that the populations from the Balkan Peninsula represent one genetically differentiated group and should be viewed as a distinct population (Fig. 1). Although the second discriminant function from the DAPC analysis differentiates populations from Croatia and Serbia as separate clusters. It showed that they are both differentiated from the population of the Pyrenees and other populations derived from the Iberian Peninsula. The analysis of *F*_*ST*_ values showed that the Griffon vulture population of Serbia is genetically most differentiated from all other populations. The population from Croatia, although genetically closest to the one from Serbia, exhibits similar *F*_*ST*_ values when compared to other native populations, but still higher than the *F*_*ST*_ values detected between all other pairs of analysed populations. This can be explained by the fact that until the nineteenth century there used to be an intermediate population in the Alps which connected the populations from Croatia and Western Europe, allowing the gene flow^25^. This, once extinct intermediate population essential for gene flow, is now being re-established through reintroduction programs in the French Alps^25^.

Even though Le Gouar^25^ presented the existence of genetic differences between the Balkan population from Croatia and those originating from the Pyrenees and Spain, the Griffon vulture population from Spain was used as a source population for the reintroduction program in Bulgaria^47^. Le Gouar^25^ explained the observed genetic differentiation as the result of the recent isolation, and not as a measure of significant long term differentiation among two distinct populations. It should be noted that the Griffon vultures which inhabit the Balkan Peninsula exhibit some specific morphological differences compared to their counterparts from the Iberian Peninsula. These could be adaptations to different climate conditions^48,49^. On average, the Griffon vultures from the Balkan Peninsula exhibit a greater body mass and have later hatching time, 1 month after the Mediterranean populations, most likely as an adaptation to a colder climate and delays of herds pasturing due to longer periods of cold^50^.

Another interesting result for the Griffon vulture population from Israel suggests that the Middle East could be recognized as a hybridization/admixture zone^26,51^, or, more likely, as the region from which European populations have originated^6^. It has been reported that young birds from the Balkan Peninsula migrate to the Middle East, where they stay between 3 and 4 years until they are sexually mature^52^, then return to the region where they have hatched, to form nests^53,54^. According to our results, it is possible that some of the birds choose to stay in the Middle East where they interbreed with the birds from the region. Another possibility is that the observed equal contribution of both genetic clusters in the Griffon vulture population from Israel suggests that it was the source population from which this species colonized the Mediterranean. It is suggested that during the Last Glacial Maximum (LGM) in Europe the European Griffon vulture populations retreated to refugia in North Africa and the Arabian Peninsula^55^. After the end of LGM the recolonization of Europe occurred in two directions: across Gibraltar into the Iberian Peninsula and across Bosporus into the Balkan Peninsula. This hypothesis is supported by the results which show that the population of Israel is the only population without a recent bottleneck. It is plausible that during the initial colonization of Europe from the Middle East the populations of the Iberian and the Balkan Peninsulas went through a founder effect and successive bottleneck, which led to the prevalence of two different genetic clusters, thus differentiating them.

Even though the two distinct groups can be recognized, AMOVA results suggest that the highest genetic variation is observed within populations and less between them. Since the within-population variance based on microsatellites is generally high, it can lead to an underestimation of variance among the populations. However, STRUCTURE analysis supports the finding that these populations highly overlap in their genetic composition, since they share two genetic clusters.

Griffon vulture species is considered to be socially monogamous and once formed couples stay together^56,57^, although rare cases of extra-pair copulation within the same colony have been observed^58^ but never confirmed by genetic analyses^59^. Furthermore, it has a wide dispersion and migrates throughout the Mediterranean. In their migrations, the birds from Serbia can be found almost exclusively in the eastern Mediterranean towards the Arabian Peninsula, but when the nesting period approaches, these birds return to the region in which they have hatched^14^. This natal philopatric behaviour is important because it can act as a mechanism for increasing genetic differences between the populations that nest in distinct regions, especially in combination with somewhat different climatic conditions. In conservation biology, this effect of philopatry is well recognized as it can be one of the causes of phylogeographic structuring due to geographic isolation caused by this kind of behaviour. This long-term isolation can be used as a major criterion to identify population, i.e. evolutionary significant unit, that deserves separate management or priority for conservation due to its high distinctiveness^60^. The effect of natal philopatry on genetic differentiation of Cinereous vulture populations from the Iberian and Balkan peninsulas was used to explain the observed genetic differences, resulting in the fact that they are now recognized as evolutionary significant units worthy of protetction^32^. New data presented in our paper show that the Griffon vulture populations from the Balkan and Iberian Peninsulas are genetically differentiated as well, and that they may have been adapted to different climatic conditions. This raises the question whether the introduction of foreign birds to the Balkan Peninsula is justified. The morphological differences, as well as the observed genetic differences^25^, should be a rationale to pay closer attention to the local populations as the source for reintroduction programs. The fact that a small percentage of introduced birds from the Iberian Peninsula to Bulgaria survived suggests that the continental Balkan population deserves different conservation strategy and that the reintroduction should not be performed with foreign birds but the ones from the already existing and viable native populations of the Balkan Peninsula. The observed differences between the populations of the Balkan and Iberian Peninsulas could be the result of a non-adaptive processes, like a bottleneck and/or a founder effect, as well as the result of the natal philopatry. In the present paper, we used selectively neutral markers which limit the conclusions about adaptive differences, enabled from the loci under selection or the morphometric data. Nevertheless, the experience of reintroduction with the birds from the Iberian Peninsula into the Bulgaria region may suggest that these differences could be the result of local adaptations as well.

## Materials and methods

### Population location and sampling

In the present study we have analysed new samples from the Griffon vulture population that inhabits the region of South-western Serbia, the gorge of the river Uvac. Uvac belongs to the protected area of the “Special nature reserve Uvac” (43°24′N 19°56′E), founded in 1971 to protect the Griffon vultures. The reserve includes three artificial lakes at altitudes from 818 to 972 m a.s.l.: Uvačko (972 m a.s.l.), Zlatarsko (876 m a.s.l.) and Radoinjsko (818 m a.s.l.). The lakes are situated in a mountainous region with steep limestone cliffs. The birds used in the present study were sampled between the years 2013 and 2019, as part of the continuous monitoring program that includes marking the young birds directly in the nests. Samplings were performed from the end of May to the beginning of June before they leave the nests. The blood samples were collected from the chicks directly in the nests and stored in sodium citrate or QUEENS buffer^61^ at – 20 °C. Blood samples were collected via venal puncture using sterile equipment with respect to animal welfare, in accordance with relevant guidelines and regulations. During the sampling, thorough morphological analysis of chicks was performed (weight, bird length, wing length, tail length, hindquarters and finger length, tarsus length, beak length, width and height) and they were tagged by the licensed personnel. For genetic analyses, we used samples from 57 different nests in order to avoid sampling of siblings. None of the birds was sacrificed or injured during the sampling process. All the procedures applied in this study were reviewed and approved by the Ministry of Nature Protection of the Republic of Serbia and the ethical committee of the Institute for Biological Research “Siniša Stanković”.

For the comparative analysis we used genetic data of the native Griffon vulture populations of Croatia (N = 37), Israel (N = 33), and the Pyrenees in France (Ossau, N = 81) published in Le Gouar^25^. The introduced populations were presented by the data from the populations of Baronies (N = 90), Causes (N = 86), Verdon (N = 41), Navaceles (N = 15) and Diois (N = 45)^25^. In the comparative analyses, we used the raw data (amplicon lengths) provided by Prof. Le Gouar (personal communication). In order to be able to compare genetic data generated in two different laboratories, six samples from the Griffon vulture population of Croatia used in the study of Le Gouar^25^ were reanalysed. The feathers from the same birds used in Le Gouar^25^ were chosen for the DNA extraction in order to be processed in the same way as the new samples from Serbia. For all analyses performed in the present work, we used data for individual birds which had at least 5 genotyped loci, thus creating the differences in the sample sizes for the same populations used in our analysis and Le Gouar^25^. In a previous study, sample sizes for Baronies, Verdon, and Diois were smaller than the sizes used in our analysis, because the authors excluded the birds of known Spanish origin from the group of these samples, which they used to create a representative group for Spain. Considering that no genetic structuring between the introduced groups and between introduced groups and the native population in Ossau was observed in Le Gouar^25^ we included these birds in the samples for Baronies, Verdon, and Diois. Furthermore, due to the fact that in the same work the low level of genetic differentiation between the samples from Ossau and Spain was observed, we dubbed the Ossau as the population of the French Pyrenees derived from the Iberian Peninsula.

### DNA extraction and amplification of microsatellite loci

The DNA extraction was performed using the GeneJET Genomic DNA Purification Kit (Thermo Fischer Scientific Cat.No. K0721). The DNA concentration and quality were checked both by a spectrophotometer (NanoPhotometer, IMPLEN, Germany) and agarose gel electrophoresis. We used ten microsatellite loci. Five were taken from Mira^36^ and the other five were chosen from Gautschi^35^ (Table 4). Microsatellite loci were amplified in PCR reactions in which forward primers were labelled with a fluorescent dye (Table 4). PCR was performed in five reactions that differed in annealing temperature (Table 4) using the following program: one cycle of initial denaturation at 94 °C for 5 min, after which there were 30 cycles of 35 s at 94 °C, 35 s at the annealing temperature (Table 4) and 35 s at 72 °C. The step of final elongation was performed at 72 °C for one hour. Loci were amplified in a 2720 Thermal Cycler (Applied Biosystems, UK) in three multiplex reactions and two individual reactions (Table 4). Amplification was performed in a volume of 20 µL with the following final concentrations of reaction components: 1 × Taq Buffer with (NH_4_)_2_SO_4_, 2.5 mM MgCl_2_, 0.8 mM dNTP mix, 1 U of reverse Taq polymerase (all components were produced by Thermo Fisher Scientific, EU) and 10 pmol of each forward and reverse primer. In order to verify the reliability of data, 10% of samples were reamplified for second time.

**Table 4:** List of loci used in genetic analyses, primers used for their amplification, fluorescent dyes used for tagging the forward primers, annealing temperature (Tm) used in each reaction for the amplification of specific microsatellite loci with the combination of loci amplified together in multiplex reactions I, II, III, and individual reactions.

Locus	Primer pairs	Label	Tm (°C)	Reference	Multiplex PCR
GF3H3	F: 5′ GTAGAATAATTTGCTCCTGG 3′	VIC	55	Mira^36^	II
	R: 5′ TGAAGGCACCTCATAGACA 3′				
GF3F3	F: 5′ GATCTTTCCCCTTCTGTG 3′	NED	55	Mira^36^	Individual PCR
	R: 5′ TTCGTGCAGTGATGCTGGTG 3′				
GF8G1	F: 5′ TGAGCAGGTGAGTCCAGAAG 3′	FAM	55	Mira^36^	II
	R: 5′ GCTCTCCTGTCATCTTGCAT 3′				
GF9C1	F: 5′ GGTGGACATTACATACACTG 3′	PET	55	Mira^36^	Individual PCR
	R: 5′ CAAGGAATCTGGACTACTAA 3′				
GF11A4	F: 5′ GATCCCTTCCAACCGAAAAT 3′	FAM	55	Mira^36^	I
	R: 5′ TGGTGACCAACGGAAGTGTG 3′				
BV11	F: 5′ TGTTTGCAAGCTGGAGACC 3′	PET	58	Gautschi^35^	III
	R: 5′ AAAAGCCTTGGGGTAAGCAC 3′				
BV12	F: 5′ TCAGGTTTTGACGACCTTCC 3′	VIC	58	Gautschi^35^	III
	R: 5′ GTGGTAACGGAGGAACAAGC 3′				
BV13	F: 5′ AAAACAGAGTTTTCACATTTTCATAAG 3′	NED	55	Gautschi^35^	I
	R: 5′ TTCAGGAAACAGAAGCATGAAC 3′				
BV17	F: 5′ TGATGTGCAGATGCGTGAC 3′	FAM	58	Gautschi^35^	III
	R: 5′ GGACTCTGATGAAGCCAAGC 3′				
BV20	F: 5′ GAACAGCACTGAACGTGAGC 3′	VIC	58	Gautschi^35^	III
	R: 5′ GTTTCTCCTGACAGTGAAATAACTC 3′				

### Fragment analysis

For fragment analysis, the first and second multiplex reactions were multipooled by the GF9C1 reaction, while the third multiplex reaction was multipooled by the GF3F3 reaction. All reactions were mixed in equal volumes and plated as one reaction in a volume of 0.5 µL. Each amplification mix contained five different loci. GeneScan 600 LIZ Size Standard was used to score alleles (Applied Biosystems, UK). Fragment analysis was performed on the 3130 Genetic Analyzer (Applied Biosystems, UK). Data were analysed using Gene Mapper Software (Life Technologies, USA).

### Population genetics

In order to assess the genetic diversity of analysed populations, we determined the following parameters for each locus and population: number of alleles, number of alleles based on a minimal sample size (obtained by rarefaction), number of private alleles based on a minimal sample size (obtained by rarefaction), observed (H_O_) and expected (H_E_) heterozygosity, random match probability and mean number of pairwise differences. Random match probability represents the parameter that expresses the probability that two randomly picked individuals from a population have a matching genotype, and is calculated as the sum of square frequencies^62^. The mean number of pairwise differences represents the measure of differences between all pairs of haplotypes in the sample. Calculations were performed using Arlequin ver. 3.5.2.2^63^ and HP-Rare 1.1^64^. The effective number of alleles per locus was calculated using the following formula: ${A}_{e}=1/\sum {p}_{i}^{2}$, where *pi* is the frequency of an *i*th allele. Arlequin software was used for assessing genetic differentiation among populations through Analysis of molecular variances (AMOVA) and estimating pairwise population and overall *F*_*ST*_ values. Statistical significance of all performed tests was assessed with 10,000 permutations. The matrix of pairwise population *F*_*ST*_ values was visualized both by R functions and two-dimensional scaling (non-metric MDS) using PAST 3.25^65^. Hardy–Weinberg equilibrium was tested using Arlequin software with 1,000,000 number of steps in MC and 100,000 dememorization steps.

In order to detect whether a significant number of targeted loci featured heterozygosity excess, we used the Wilcoxon signed-rank test implemented in the software Bottleneck v.1.2.02^66^ this analysis was used, since excessed heterozygosity would imply a recent bottleneck event. The two-phase mutation model (TPM)^67^ was used and parameterized to 25% of stepwise mutation. The effect of the recent bottleneck was also evaluated based on the Garza–Williamson index (G–W) which represents the ratio between the number of alleles and allelic range and was calculated using Arlequin software. Linkage disequilibrium between the pairs of loci was estimated using the likelihood ratio test in Arlequin software with 10,000 numbers of steps in MC and 10,000 dememorization steps. Effective population sizes for the analysed populations were calculated with NeEstimator 2.1^68^ using the linkage disequilibrium method and monogamy mating model. In order to correct the probabilities when multiple tests were done simultaneously, we performed a sequential Bonferroni test for all performed analyses^69^.

### Population structure

Clustering of the analysed populations and the observed distances among samples were presented using discriminant analysis of principal components (DAPC)^70^ using the R package adegenet^71^. This method consists of performing linear discriminant analysis (LDA) on the principal components analyses (PCA) transformed matrix. The number of retained PCs was estimated using randomly repeated cross-validation (100 iterations) which consisted of performing DAPC on 90% of randomly sampled training set observations (stratified sampling was used so that the training set consisted of 90% of the observations from each population) after retaining 10–83 PCs and using the obtained models to predict the groups (populations) in the remaining 10% of samples (test set). Average prediction success per group was used as the metric to choose the optimal model.

In order to estimate the number of genetic clusters represented in the native populations of the Griffon vulture we used the STRUCTURE v 2.3.4 software^72–75^. We also performed the same analysis including the reintroduced populations. The analysis was performed using the admixture model with a burn length of 10,000 and a Markov Chain Monte Carlo (MCMC) of 100,000 randomizations. The range of the possible number of clusters (K) was from 1 to 10, with a series of 10 runs for each K. STRUCTURE harvester (Web v0.6.94, https://taylor0.biology.ucla.edu/structureHarvester/^76^) was used to analyse the results obtained by STRUCTURE. Based on the results generated by STRUCTURE this software creates a plot of the mean likelihood value per *K* value and calculates the highest value of the second-order rate of change (Delta K) according to the method of Evanno^77^ in order to detect the number of K groups that best fits the data set. The model choice criterion, LnP(D), implemented in the STRUCTURE which detects the true *K* as an estimate of the posterior probability of the data for a given *K* was evaluated as well. The most likely scenario was chosen and used to graphically plot both the analysed individuals and populations.

## Supplementary information

Supplementary Figures.

Supplementary Tables.
