# Supplementary material for: Genetic diversity of the Griffon vulture population in Serbia and its importance for conservation efforts in the Balkans
Source: Sci Rep. 2020 Nov 23;10:20394. doi: 10.1038/s41598-020-77342-1 (PMC7684298; doi:10.1038/s41598-020-77342-1)
Supplement: Supplementary file 1 — Supplementary Figures. [file 41598_2020_77342_MOESM1_ESM.pdf]

**Genetic diversity of the Griffon vulture population in Serbia and its importance for conservation efforts in the Balkans**

Slobodan Davidović[1], Mihailo Jelić[2], Saša Marinković[3], Milica Mihajlović[4], Vanja Tanasić[4], Irena Hribšek[5], Goran Sušić[6], Milan Dragićević[7], Marina Stamenković-Radak[1, 2]

[1]Department of Genetics of Populations and Ecogenotoxicology, Institute for Biological Research „Siniša Stanković" – National Institute of Republic of Serbia, University of Belgrade, Bulevar Despota Stefana 142, 11060 Belgrade, Serbia

[2]University of Belgrade-Faculty of Biology, Studentski trg 16, 11000 Belgrade, Serbia

[3]Department of Ecology, Institute for Biological Research „Siniša Stanković" – National Institute of Republic of Serbia, University of Belgrade, Bulevar Despota Stefana 142, 11060 Belgrade, Serbia

[4]Center for Forensic and Applied Molecular Genetics, Faculty of Biology, University of Belgrade, Studentski trg 16, 11000 Belgrade, Serbia

[5]Birds of Prey Protection Foundation, Bulevar Despota Stefana 142, 11060 Belgrade, Serbia

[6]Ornithological station Rijeka, Croatian Academy of Sciences and Arts, Ružićeva 5/2, 51000 Rijeka, Croatia

[7]Department of Plant Physiology, Institute for Biological Research „Siniša Stanković" – National Institute of Republic of Serbia, University of Belgrade, Bulevar Despota Stefana 142, 11060 Belgrade, Serbia

**Corresponding author:** Slobodan Davidović, Bulevar Despota Stefana 142, 11060 Belgrade, Serbia, e-mail: slobodan.davidovic@ibiss.bg.ac.rs; ORCID: 0000-0001-9317-6870

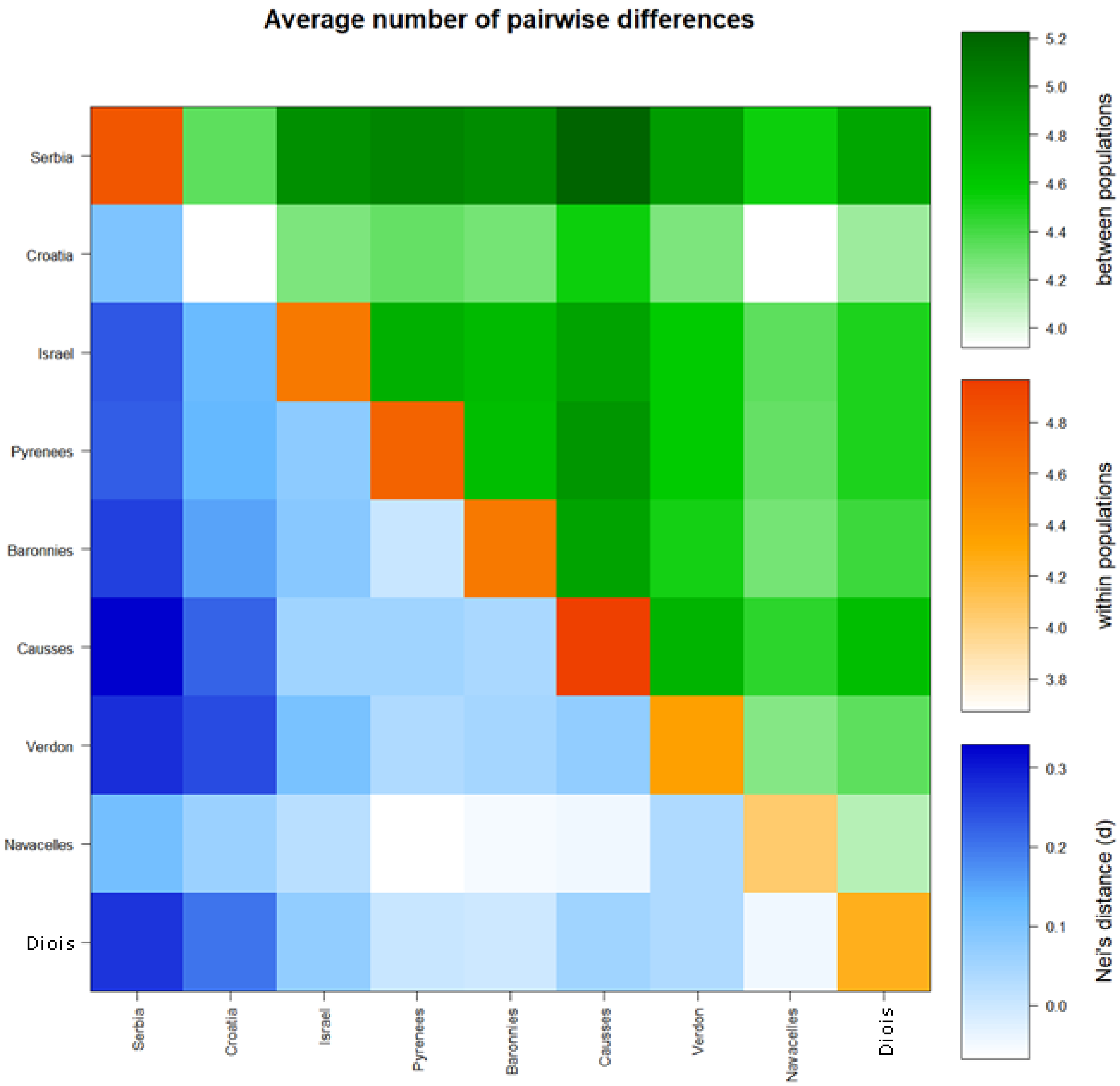


**Figure S1** Matrix of the average number of pairwise differences and Nei's distances detected in native and introduced *G. fulvus* populations based on the analysis of microsatellite loci. The average number of pairwise differences between populations is presented above diagonal, the average number of pairwise differences within the population is presented diagonal and Nei's distances are presented below diagonal. The average number of pairwise differences are presented in Table 1.

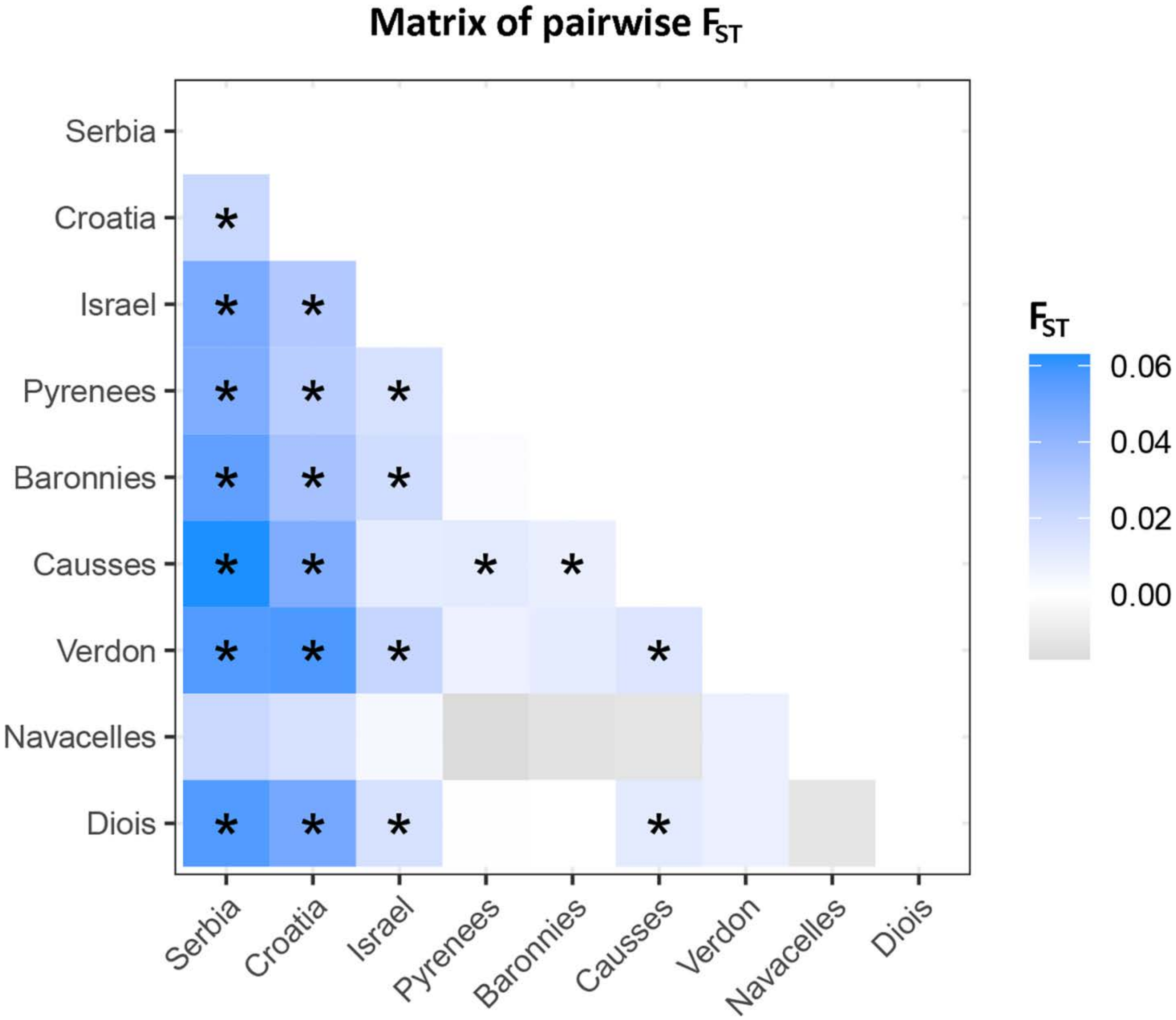


**Figure S2** Matrix of pairwise $F_{ST}$ distances between the *G. fulvus* population of Serbia and other native and introduced *G. fulvus* populations based on the analysis of 10 microsatellite loci. Statistically significant $F_{ST}$ values are marked with an asterisk (*). Population pairwise $F_{ST}$ values are presented in Table 3.

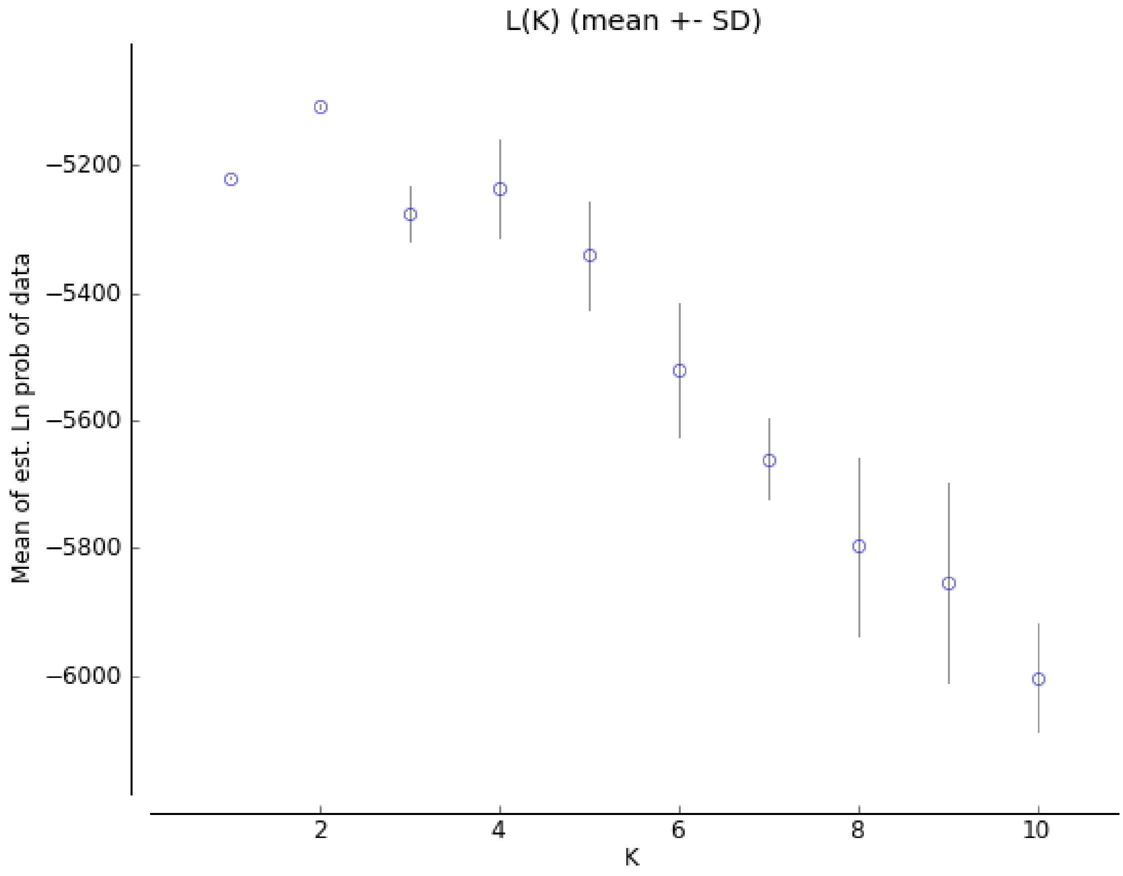


**Figure S3** Ln values of probability for the assumed number of genetic clusters.

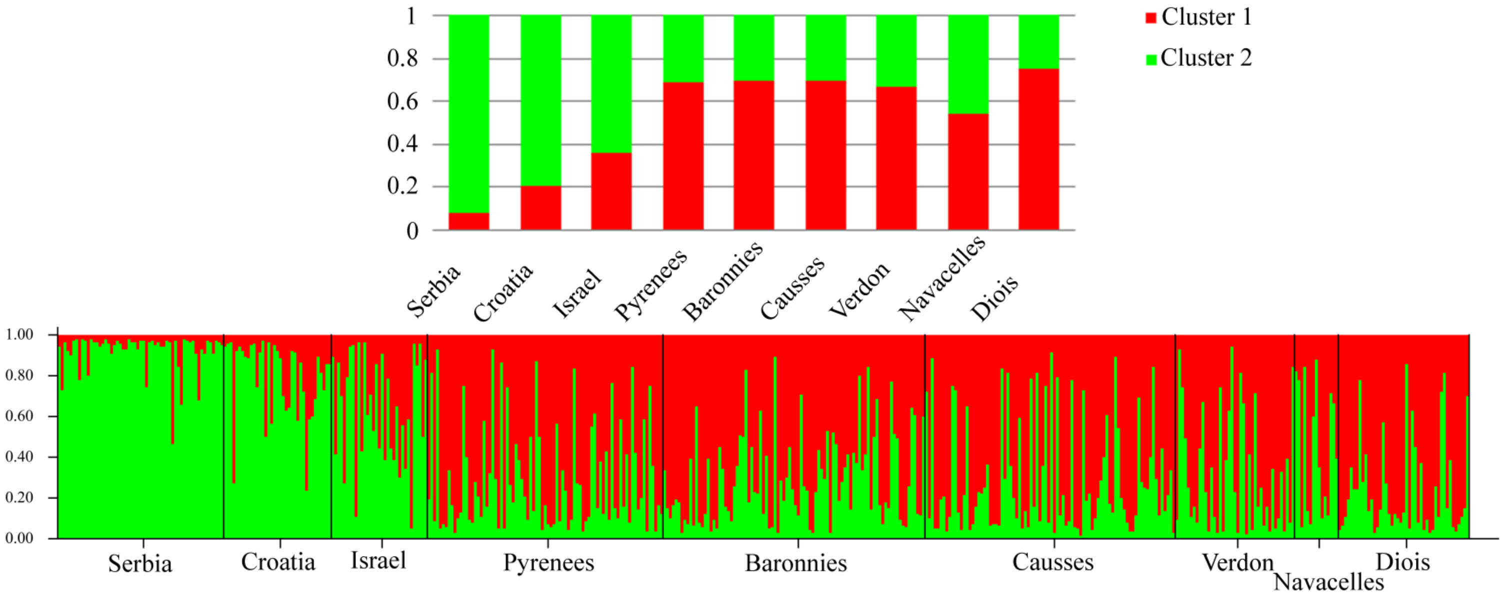


**Figure S4** (a) Proportions of inferred STRUCTURE clusters (*K*=2) from the native and introduced populations. (b) Proportions of the inferred STRUCTURE clusters (*K*=2) from the individuals.
